# Expansion of discharge planning system in Japan: Comparison of results of a nationwide survey between 2001 and 2010

**DOI:** 10.1186/1472-6963-12-237

**Published:** 2012-08-03

**Authors:** Satoko Nagata, Hikari Tomura, Sachiyo Murashima

**Affiliations:** 1 Department of Community Health Nursing, Graduate School of Medicine, The University of Tokyo, 7-3-1 Hongo, Bunkyo, Tokyo, 113-0033, Japan; 2 Department of Nursing Sciences, Tokyo Metropolitan University, 7-2-10, Higashi-Ogu, Arakawa, Tokyo, 116-8551, Japan; 3 Oita University of Nursing and Health Sciences, 2944-9 Megusuno, Oita City, Oita, 870-1201, Japan

**Keywords:** Discharge planning, Hospital administration, Length of stay, Nationwide survey, Quality of health care

## Abstract

**Background:**

In response to the rapid aging of the population in Japan, many care systems have been created in quick succession. Establishment of discharge planning departments (DPDs) in hospitals is one of them. In this study, we compared the distribution and characteristics of DPDs and the characteristics of the hospitals that have DPDs between 2001 and 2010 in Japan.

**Methods:**

We mailed a questionnaire about the characteristics of hospitals and existence and situation of DPDs to all general hospitals with 100 or more general beds in 2001 and in 2010.

**Results:**

In 2001, of the 3,268 hospitals queried, 1,568 (48.0%) responded and 1,357 (41.5%) were selected for data analysis. In 2010, among 2,600 hospitals, 940 hospitals (36.1%) responded and 913 (35.1%) met the inclusion criteria. The percentage of hospitals with DPDs increased from 30% to more than 70% between the two surveys. More departments were under the direct control of the hospital director and more physicians participated in discharge planning activities in 2010 than in 2001. In 2001, private hospitals and hospitals with an affiliated institution or agency tended to have a DPD; however, the relationship between these factors and the presence of a DPD had disappeared in 2010. Larger hospitals and hospitals with more nurses per patient tended to have a DPD both in 2001 and 2010.

**Conclusions:**

Since 2008, the establishment of a DPD has been directly connected to medical fees so hospital administrators might have recognized the DPD as a “necessary and paid for” department. Having a DPD was the majority’s policy in Japan, and we must recognize the importance of quality assurance through DPDs from now on, especially in small hospitals.

## Background

Japan’s universal and egalitarian health care system has helped to keep its population healthy at an exceptionally low cost. Global health indices such as life expectancy at birth in Japan are among the best in the world, while its health expenditure is fairly low [1]. However, how health care costs will be financed is one of the most daunting challenges for Japan. Although the bulk of health expenditures are financed by social insurance premiums, a quarter of health expenditures comes from the central government’s general revenues. Increasing health expenditures due to the ageing society and advances in medical technology impose pressures on the nation's finances. The Japanese government must control total health expenditures to contain the overall budget [2].

In Japan, the average length of hospital stay has been much longer than in other developed countries [3]. Economic incentives to decrease the length of stay are now incorporated into medical payment schedules. In addition, the new flat-free payment system called the Diagnosis Procedure Combination (DPC) was introduced in 2003 [4]. The DPC-based payment system is divided into a DPC component and a fee-for-service component. The amount of payment per day in the DPC component decreases gradually at three levels of days in hospital [4], so that hospitals have the incentive to shorten the length of stay. Such efforts have shortened the average length of stay in general beds from 23.5 days in 2001 to 18.2 days in 2010 [3].

To achieve smooth transfer of patients after the acute phase to another facility or to home for recuperation in as short a period of time as is considered safe, discharge planning is important. Discharge planning has been gaining special attention as a way to ensure continuity of high-quality patient care and to save health care costs by preventing over-long hospitalizations and/or unnecessary re-hospitalizations. In the United States, since 1986 hospital Medicare participation has required discharge planning [5]. Many researchers have pointed out the effectiveness of discharge planning, citing outcomes such as shorter hospitalizations, greater satisfaction of clients, more usage of home care services and fewer readmissions [6-10].

In Japan, discharge planning has been encouraged within the framework of medical care insurance. Actually, “forming a guidance plan for discharge” has been included in requirements to receive acute care hospital fees since 2002. In addition, a premium fee is paid to hospitals that hold a conference with a community care worker before a patient’s discharge. Furthermore, since 2008, discharge planning for older patients, psychiatric patients, and patients who need long-term care has been part of the medical treatment fee schedule. One requirement for hospitals to receive this new discharge planning reimbursement fee is the creation of a Department of Discharge Planning in each hospital. Under such circumstances, establishment of these departments is expected to increase among hospitals all over Japan, although much is not known about matters related to discharge planning departments (DPDs), such as their exact number, the types of hospitals that tend to establish DPDs, etc.

We investigated discharge planning systems at all hospitals with 100 or more general beds in Japan and the variables related to the establishment of DPDs in 2001 [11,12]. The percentage of hospitals with a DPD was 29.4%. Hospital size, hospital type, nurse/patient ratio, establishing body, and affiliated institutions were related significantly to the status of the establishment of DPDs [11]. In addition, we found that hospitals with a DPD performed more discharge planning activities for patients than hospitals without such a department [12]. This result indicates that the presence of a DPD enhances a hospital’s activity in relation to discharge planning.

Japanese hospitals vary in the number of beds and in the type of establishing body. It might be useful to clarify the variables related to the establishment of DPDs, not only to encourage effective discharge planning systems but also to provide information on the process of formation of these pioneering systems. Therefore, we compared the situation regarding DPDs between 2001 and 2010. For this purpose, in 2010 we performed a survey similar to that performed in 2001. By comparison of survey results between 2001 and 2010, we intended to clarify how an innovative change such as the establishment of DPDs was distributed throughout the whole country.

The objectives of this paper are to compare the distribution of DPDs and characteristics of the hospitals that have DPDs between 2001 and 2010 in Japan.

## Methods

### Subjects and procedure

Criterion for inclusion in the study sample was a general hospital with 100 or more beds for acute care. Psychiatric hospitals or facilities for the disabled were excluded because the discharge process for patients in those facilities differed from that for acute care patients. We identified all such hospitals using the “Hospital Catalogue 2001-2002” [13] in 2001 and the database of the Japan Medical Press, Inc. [14] in 2010. Both were among the most reliable databases for Japanese hospitals throughout Japan. The total number of target hospitals was 3,268 in 2001 and 2,600 in 2010.

We mailed a questionnaire to the director of the nursing service department in November 2001 and April 2010**.** Additional file 1 is the questionnaire sent in 2001, and Additional file 2 is the questionnaire sent in 2010. Either the director of nursing or the person in charge of discharge planning was to answer the questionnaire. In a cover letter we stated clearly that the information about each hospital would not be identified publicly and that confidentiality would be maintained at all times. The return of the completed questionnaire implied informed consent. This study was approved by the Ethics Committee of the Graduate School of Medicine, University of Tokyo (reference number is 2959).

### Discharge planning and discharge planning departments

For this report, we have defined “discharge planning” as a process to identify and confirm arrangements with other hospitals, institutions, or home care agencies that can meet both patients’ and families’ needs after discharge from the initiating hospital and to provide support necessary for discharge, such as information, guidance and coordination of services to patients, families and service providers. The definition of DPD for this report is the “department in the hospital to which the staff in charge of discharge planning belongs.”

DPDs in the US are commonly referred to as departments of case management, social work, care coordination, utilization review, or patient/family services. The professionals working in these departments are commonly nurses or social workers. The role of a team member can vary from hospital to hospital, such as case managers, utilization review persons, care coordinators, clinical documentation specialists and social workers [15].

In Japan, the name and roles of the department also vary, and most of the staff of DPDs is comprised of nurses and social workers, the same as in the US [11]. However, clerical staff is in charge of utilization review in Japan, and usually the department for utilization review is different from the DPD. In addition, both nurses and physicians record patient information and the information management section is different from the DPD. As a result, the main roles of the DPD in Japan are as care coordinators and social workers.

### Variables

#### Characteristics of hospitals

The questionnaire included items about hospital characteristics such as the establishing body, number of beds as the variable for hospital size, types of affiliated organizations or institutions, and average length of hospitalization for general beds.

As to the number of patients per nurse, the standard for medical reimbursement was changed in 2006. The previous standard was defined by the number of inpatients per employed nurses; the new standard is defined by the number of inpatient per working nurses per working hour. For example, if there were 20 nurses assigned to a ward with 40 patients, according to the previous standard it was calculated that there was one nurse for every 2 patients (=2:1). On the other hand, if there are 20 nurses assigned to a ward, at most only 4 nurses can work at same time because of shift work; therefore, by the new standard it was calculated that 40:4 = 10:1. One nurse for 2 patients, for 2.5 patients, for 3 patients, and for 3.5 patients in the previous standard corresponds to one nurse for 10 patients, for 13 patients, for 15 patients and for 18 patients, respectively. In addition, a new nursing allocation of “7 patients per nurse” was established for more advanced care (which corresponds to 1.4 patients according to the old standard). In this paper, we indicate nurse staffing for both 2001 and 2010 using the new standard, i.e. 7:1, 10:1, 13:1 and 15:1 or more (which means 1.4, 2, 2.5, and 3 and more according to the old standard).

#### System for discharge planning

We asked about the presence of “a department for discharge planning”. The definition of “department for discharge planning” for this report was “department in the hospital to which staff in charge of discharge planning belongs.” In addition, we elicited information about the DPD; the year of establishment, profession of the person in charge, composition of staff according to discipline (medical social worker, nurse/public health nurse, clerical staff, and physician), and services provided separate from discharge planning.

### Statistical analysis

At first, we compared the characteristics of DPDs and characteristics of the hospitals between the two years by the Chi-squared test and Mann–Whitney U test. Next, we checked the relationship between hospital characteristics and existence of a DPD by bivariate analysis. After controlling for multicollinearity, logistic regression analysis was done to investigate the factors related to the existence of DPDs. SPSS ver. 17 was used.

## Results

### Response rate and sample

**Figure 1:**
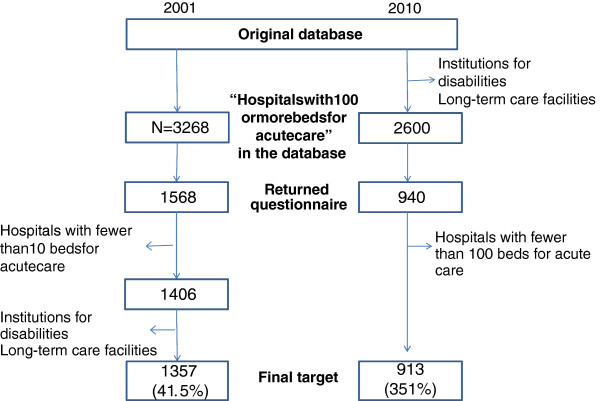
Process of sampling of target hospitals.

The process of sampling of target hospitals is shown in Figure 1. In 2001, from a population of 3,268 hospitals, 1,568 (48.0%) responded. However, 162 hospitals had fewer than 100 general beds, and thus did not meet the inclusion criteria for the study. In addition, the remaining 1,406 hospitals included institutions for the disabled and other long-term care facilities, which were not suitable for the purpose of this study. To ensure a uniform sample, we established an additional criterion, “average length of hospital stay of less than 90 days”, as one of the criteria to compute the standard fee for acute care hospitals. Ultimately, 1,357 (41.5%) hospitals were selected for data analysis.

In 2010, learning a lesson from the experience in 2001, we excluded institutions for disabilities and that had long-term care facilities from the original database. As a result, 2,600 hospitals were surveyed. Of these, 940 hospitals (36.1%) responded to the survey. When the response indicated that a hospital had less than 100 beds or no information was provided about the situation of a DPD, the hospital was excluded. As a result, the number of target hospitals was 913 (35.1%).

### Comparison of hospital characteristics between 2001 and 2010

The characteristics of the target hospitals are shown in Table 1.

**Table 1:** Characteristics of target hospitals

	**Year 2001**		**Year 2010**		**p-value**
	**Number**	**Percent**	**Number**	**Percent**	
Total	1357	100.0	913	100.0	
Establishing bodies					
Public sector	722	53.2	373	40.9	<0.001^a,c^
Municipality	400	29.5	234	25.6	
Public association	178	13.1	83	9.1	
National government	144	10.6	56	6.1	
Private sector	635	46.8	534	58.5	
Non-profit medical corporation	414	30.5	288	31.5	
Other	221	16.3	246	26.9	
Type of hospital					
Having beds for long-term care	223	16.4	201	22.0	<0.001^a^
Community care support hospital	16	1.2	313	34.3	<0.001^a^
Special function hospital	49	3.6	57	6.2	0.004^a^
Affiliated institution or agency					<0.001^a,d^
Having institution or agency	572	42.2	482	52.8	
Home care agency	507	37.4	356	39.0	
Institution for elderly	239	17.6	197	21.6	
No affiliated institution or agency	743	54.8	431	47.2	
Missing data	42	3.1	0	0.0	
Number of hospital beds					
100-199	507	37.4	333	36.5	
200-399	512	37.7	338	37.0	
400 or more	326	24.0	242	26.5	
Missing data	12	0.9	0	0.0	
mean (SD)	312.2 (203.3)		321.5 (200.6)		ns^b^
Average length of hospital stay					
Less than 20 days	462	34.0	724	79.3	
20-30 days	654	48.2	116	12.7	
30 days or more	203	15.0	46	5.0	
Missing data	38	2.8	27	3.0	
mean (SD)	24.1 (10.3)		17.8 (8.9)		<0.001^b^
Number of patients per nurse in each hospital^e^					
7:1	0	0.0	438	48.0	<0.001^b^
10:1	527	38.8	395	43.3	
13:1	561	41.3	35	3.8	
15:1 or more	254	18.7	42	4.6	
Missing data	15	1.1	3	0.3	

ns: not significant.

^a^ Chi-squared test. ^b^ Mann-Whitney U test.

^c^ P-value for public sector (total) vs. private sector (total).

^d^ P-value for having institution or agency (total) vs. no affiliated institution or agency.

^e^ Items for year 2001 correspond to values for new standard beginning 2006.

As to the establishing body, in 2001 the majority was established by the public sector (53.2%) whereas in 2010 the majority (58.5%) was established by the private sector (p < 0.001). Within the private sector, non-profit medical corporations were the most prevalent establishing body in both periods.

Hospitals with beds for long-term care increased from 16.4% in 2001 to 22.0% in 2010 (p < 0.001). Community care support hospitals showed the greatest increase from 2001 to 2010 (1.2% and 34.3%, respectively p < 0.001). The percentage of special function hospitals also increased (3.6% in 2001, 6.2% in 2010, p = 0.004). Average number of hospital beds was 300 or more, with no difference observed between the two time points. The average length of hospital stay was 17.8 days in 2010, which is much shorter than the 24.1 days in 2001 (p < 0.001). The percentage of hospitals with an affiliated institution or agency increased significantly (42.2% in 2001, 52.8% in 2010, p < 0.001). As to the standard for the number of patients per nurse in each hospital, it was revealed that the nursing staff was increased in 2010.

### Comparison of situations related to DPDs between 2001 and 2010

Percentage of hospitals with a DPD increased greatly between the two time points (29.4% in 2001, 73.2% in 2010). As to the establishment of a DPD, data from the 2010 survey showed that 81 hospitals had formed a department by 1999 while 173 hospitals did so between 2000 and 2004, 136 between 2005 and 2007, and 180 after 2008. Cumulative percentage of hospitals with discharge departments was 8.5% by 1999, 17.1% by 2002, 32.5% by 2005, and 51.4% by 2008.

Information related to DPDs is shown in Table 2. As to characteristics of the DPDs, their position in the hospital changed between the two periods examined. For example, the percentage of departments under the direct control of the hospital director was increased between the two periods from 11.5% to 25.1% (p < 0.001). In 2010, about 40% of persons in charge were physicians, which was a large increase from 2001 (23.1% in 2001, 39.2% in 2010, p < 0.001). As to staff, those in all job categories significantly increased between 2001 and 2010. Regarding services provided by the department other than discharge planning, visiting nurse services and home care instructions for outpatients decreased (p < 0.001), and advice on availability of appropriate outpatient services increased (p = 0.003).

**Table 2:** Rate of establishment and characteristics of discharge planning departments

	**Year 2001**		**Year 2010**		**p-value**
	**Number**	**Percent**	**Number**	**Percent**	
Total	1357	100.0	913	100.0	
Department for discharge planning					
Present	399	29.4	668	73.2	<0.001
Absent	958	70.6	245	26.8	
Discharge planning department characteristics					
Total	399	100.0	668	100.0	
Establishment year					
~1989	77	19.3	37	5.5	NA
1990~1994	45	11.3	16	2.4	
1995~1999	141	35.3	28	4.2	
2000~2004	95	23.8	173	25.9	
2004~	-		316	47.3	
Position in hospital					<0.001
Administrative division	127	31.8	186	27.8	
Nursing division	95	23.8	89	13.3	
Others	75	18.8	100	15.0	
Under direct control of hospital director	46	11.5	168	25.1	
Clinical division	44	11.0	97	14.5	
Person in charge					<0.001
Medical social worker	116	29.1	148	22.2	
Nurse/Public health nurse	110	27.6	176	26.3	
Physician	92	23.1	262	39.2	
Clerical staff	59	14.8	65	9.7	
Staff composition (multiple answers)					
Medical social worker	217	54.4	548	82.0	0
Nurse/Public health nurse	162	40.6	507	75.9	<0.001
Clerical staff	99	24.8	428	64.1	<0.001
Physician	77	19.3	305	45.7	<0.001
Service other than discharge planning* (multiple answers)					
Responding to community healthcare workers	319	79.9	500	74.9	ns
Advice on availability of appropriate outpatient services	292	73.2	502	75.1	0.03
Consultation on matters related to medical fees	279	69.9	419	62.7	ns
Home care instructions for outpatients	219	54.9	240	35.9	<0.001
Visiting nurse	111	27.8	86	12.9	<0.001

Chi-squared test. ns: not significant, NA: not applicable.

* Services provided separately from discharge planning.

### Variables related to DPDs in 2001 and 2010

At first, bivariate analysis was done in order to find factors related to the existence of DPDs by year. In 2001, being a special function hospital, having an affiliated institution or agency, higher number of hospital beds, shorter length of stay, and fewer patients per nurse were significantly related to the existence of a DPD. In 2010, public sector hospitals, special function hospitals, not having beds for long-term care, a greater number of hospital beds, and fewer patients per nurse were related to the existence of DPDs with significance. Among these factors, in the logistic regression analysis the variables of special function hospital, having beds for long-term care, and length of hospital stay were omitted because they were strongly related to the number of patients per nurse. As a result, establishing bodies, number of hospital beds, number of patients per nurse, and existence of an affiliated institution or agency were used for logistic regression analysis.

The results of logistic analysis are shown in Table 3. Number of beds was strongly related to the presence of a DPD in both years examined. Also the number of patients per nurse was related to the presence of such a department; however, the relationship was stronger in 2001 than in 2010. Though the existence of a DPD was related significantly to being a private sector hospital and having an affiliated institution or agency in 2001, these relationships disappeared in 2010.

**Table 3:** Variables related to the presence of discharge planning departments in 2001 and 2010 -logistic regression analysis

	**Year 2001**				**Year 2010**			
	**Odds ratio**	**95% Confidential Interval of Odds ratio**		**P-value**	**Odds ratio**	**95% Confidential Interval of Odds ratio**		**P-value**
		**Low**	**High**			**Low**	**High**	
Establishing bodies (ref. =public sector)								
Private sector	1.546	1.169	2.044	0.002	0.741	0.524	1.048	0.090
Number of general beds (ref.=100-199)								
200-399	1.669	1.231	2.263	0.001	2.273	1.600	3.228	0.000
400-	2.589	1.797	3.730	0.000	4.033	2.494	6.521	0.000
Number of patients per nurse(ref.=10)								
7	-			1.343	0.950	1.900	0.095	
13	0.651	0.493	0.860	0.003	0.690	0.336	1.420	0.314
15 or more	0.445	0.299	0.663	0.000	0.494	0.253	0.963	0.038
Affiliated institution or agency (ref.=none)								
Exists	1.462	1.122	1.904	0.005	1.036	0.746	1.438	0.832

## Discussion

### Response rate and characteristics of target hospitals

The response rate was lower in 2010 than in 2001. In 2010, the questionnaires were distributed in March, which is the end of the fiscal year in Japan, so clerical staff might have been busier than at other times. In 2001, the questionnaires were distributed in November. Among responding hospitals, the percentage of private sector hospitals was increased in 2010. According to the data of Ministry of Health Labor and Welfare [16], the percentage of public sector hospitals decreased from 31.6% in 2001 to 29.5% in 2010, so our results may reflect this tendency. Average length of hospital staywas much shorter in 2010 than in 2001. This finding was supported by Japanese national data [3] showing that the average length of stay in general beds in all of Japan became shorter from 2001 to 2010.

Increase in DPDs from 2001 to 2010.

The percentage of hospitals with a DPD had increased greatly by 2010. According to the diffusion of innovation theory, innovators and early adopters represent 16% of a population and early majorities are 34% [17]. Our survey data from 2010 regarding the year of establishment of DPDs suggested that more than 16% of the hospitals had established such a department by 2002 and that more than 50% had established a department by 2008. The establishment of DPDs moved from the early adopter level to the early majority level through the initiation of the Long-term Care Insurance System in 2000 and by the obligation to form a guidance plan for discharge from an acute care hospital since 2002, and approached the late majority level by setting the discharge planning fee based on the requirement for creation of a DPD. This means that the establishment of a DPD was the majority’s policy, and we have to recall the importance of quality assurance during this stage.

### Situation of DPDs in 2001 and 2010

As to the position of DPDs in hospitals, direct control by the hospital director had increased by 2010. Also, as to the person in charge of the department, the role of physicians was increased by 2010. Beginning in 2008, the establishment of such a department was directly connected to medical fees, so the hospital administrators might have recognized the DPD as a “necessary and paid for” department. As to staff allocation, the number of professionals in each category increased from 2001 to 2010, which means departments having multiple professionals had increased. This tendency is effective for adequate discharge planning [15,18].

### Variables related to presence of DPDs in 2001 and 2010

In 2001, there was a relationship between the existence of DPDs and private hospitals and hospitals with an affiliated institution or agency; however, that relationship had disappeared in 2010. Hospitals with affiliated long-term care facilities or home care agencies tend to connect strongly with the community. Therefore, more of such hospitals established discharge planning. In addition, as private hospitals could try new systems freely without restriction, they were able to play the role of innovator. However, DPDs were distributed widely and became a requirement for discharge planning fees, so that the number of such departments increased at an accelerated pace. In addition, as to establishing bodies, changes in the economic status encouraged privatization of national or local municipal institutions in Japan. As a result, the public sector hospitals also have to take into account financial issues because of privatization so they have undertaken discharge planning tasks.

On the other hand, the number of beds was related to the presence of DPDs in both periods. It is difficult to establish specific departments in small hospitals. A previous study revealed that the existence of a DPD was related to more discharge planning activities [12]. On another front, some hospitals without a DPD made tight connections with community resources to assure the quality of discharge planning. It is a future task for small hospitals to provide necessary discharge planning for patients.

In both years, the hospital whose number of patients per nurse was small tended to have a DPD. A hospital with abundant staffing might be in charge of more severely ill patients so the needs of discharge planning might be large. In addition, well-staffed hospitals might be better able to undertake careful planning and provide meticulous services in contrast to hospitals with unmet staffing needs.

### Limitation

The composition of the sample was not similar between 2001 and 2010, perhaps because of the difference in response rate and change in the social situation. Our data were anonymous and we were not able to match responders in the two databases.

The total quality of discharge planning in hospitals is not decided only by the presence of a DPD. In some hospitals, primary nurses take the role of discharge planner. On the other hand, in some hospitals the function of discharge planning may be insufficient even if a DPD exists. Evaluation of the quality of discharge planning by DPD should be the next focus for this research.

## Conclusion

The survey was performed to determine the situation surrounding the establishment of DPDs among all hospitals having more than 100 general beds in Japan in 2001 and 2010. The percentage of establishment of such departments increased from 30% to more than 70%. More departments were under the direct control of the hospital director and more physicians participated in discharge planning activities in 2010 than in 2001. Since 2008, the establishment of such departments was directly connected to medical fees so that hospital administrators might have recognized the DPD as a “necessary and paid for” department. In 2001, private hospitals and hospitals with an affiliated institution or agency were related to having a DPD; however, those relationships had disappeared in 2010. The reimbursement of medical fees might encourage the distribution of DPDs. On the other hand, many small hospitals could not manage such a department, so there must be discussion of quality assurance in discharge planning for all hospitals in the country.

## Abbreviation

DPD, Discharge planning department.

## Competing interests

The authors declare that they have no competing interests.

## Authors’ contributions

All authors contributed to the conception and design of the study and the acquisition of data. SN analyzed data and all authors contributed to interpretation of data. All authors contributed to drafting the article or revising it critically for important intellectual content, and final approval of the version to be submitted. All authors read and approved the final manuscript.

## Pre-publication history

The pre-publication history for this paper can be accessed here:

http://www.biomedcentral.com/1472-6963/12/237/prepub

## Supplementary Material

Additional file 1 **Survey about the situation of discharge planning at all hospital in Japan (in 2001) originally in Japanese.** (DOC 126 kb). Click here for file

Additional file 2 **Survey about the situation of discharge planning at all hospital in Japan (in 2010) originally in Japanese.** (DOC 58 kb). Click here for file
