# Supplementary material for: Expansion of discharge planning system in Japan: Comparison of results of a nationwide survey between 2001 and 2010
Source: BMC Health Serv Res. 2012 Aug 3;12:237. doi: 10.1186/1472-6963-12-237 (PMC3444405; doi:10.1186/1472-6963-12-237)
Supplement: Additional file 1 — Survey about the situation of discharge planning at all hospital in Japan (in 2001) originally in Japanese. (DOC 126 kb). [file 1472-6963-12-237-S1.doc]

**Survey about the situation of discharge planning at all hospital in Japan (in 2001)**

originally in Japanese

**I. Overview of your hospital**

Q1. Establishing body of your hospital

1. Nation (Ministry of Health, Labour and Welfare)

2. Nation (Ministry of Education、Culture, Sports, Science and Technology)

3. Nation (Others)

4. Prefecture

5. Municipality

6. Japanese Red Cross Society

7. Social Welfare Organization Saiseikai Imperial Gift Foundation

8. Koseiren

9. Social insurance bodies

10. Medical corporation

11. Social welfare corporation

12. Educational corporation

13. Company

14. Individual

15. Others

Q2. Number of bed of your hospital

Total number: _____beds

Number of general bed: _____beds

Number of bed for long-term care: _____beds

Q3. Type of your hospital (multiple answers)

1. General hospital

2. Community care support hospital

3. Special functioning hospital

4. Having beds for long-term care (for long-term care insurance)

5. Having beds for long-term care (for medical insurance)

6. Hospital for elderly

7. Rehabilitation hospital

8. Others

*Q4. Omitted because the results were not used the analysis in this article*

Q5. Affiliated institution or agency of your hospital

1. None

2. Home care support center

3. Visiting nurse service station

4. Home helper station

5. Healthcare facility for the elderly

6. Welfare facilities for the elderly

7. Care house

8. Others

*Q6. Omitted because the results were not used the analysis in this article*

Q7. Average length of stay in your hospital in last year: _____days

Q8. Number of patients per nurses based on the basic hospitalization fee standard: ____ /1 nurse

*Q9. Omitted because the results were not used the analysis in this article*

**II. Patients’ discharge situation**

*Q10-14. Omitted because the results were not used the analysis in this article*

Q15. In your hospital, does the discharge planning department exist?

1. Yes -> Jump to Section III, Q19

2. No

For the respondent answering “no”

- Q16 The reason of non-existence of discharge planning department

Q17 Future plan for establish an discharge planning department

Q18 Your idea or problem for discharge planning

- Thank you for your cooperation.

III. Discharge planning department system

Q19. Answer the name of your department and establishment year: _______

Q20. The department’s position in your hospital

1. Clinical division

2. Nursing division

3. Administrative division

4. Under direct control of hospital director

5. Others

Q21. Service of the department (multiple answers)

　　1. Only discharge planning

2. Service about medical expenditure

3. Home care instructions for outpatients

4. Service about medical consultation

5. Visiting nurse

6. Responding to community healthcare workers

7. Others

Q22 Write down each staff’s number

1. Physician: ____

2. Nurse: ____

3. Public health nurse: ____

4. Medical social worker: ____

5. Clerical staff: ____

6. Others: ____

*Q23-24. Omitted because the results were not used the analysis in this article*

**IV. Activities of discharge planning**

*Omitted because the results were not used the analysis in this article*

Thank you for your cooperation.
