# Supplementary material for: Expansion of discharge planning system in Japan: Comparison of results of a nationwide survey between 2001 and 2010
Source: BMC Health Serv Res. 2012 Aug 3;12:237. doi: 10.1186/1472-6963-12-237 (PMC3444405; doi:10.1186/1472-6963-12-237)
Supplement: Additional file 2 — Survey about the situation of discharge planning at all hospital in Japan (in 2010) originally in Japanese. (DOC 58 kb). [file 1472-6963-12-237-S2.doc]

**Survey about the situation of discharge planning at all hospital in Japan (in 2010)**

originally in Japanese

**I. Overview of your hospital**

Q1. Establishing body of your hospital

1. Nation

2. Prefecture and Municipality

3. Other public institution

4. Medical corporation

5. Public benefit corporation

6. Educational corporation

7. Company

8. Other corporation

9. Individual

10. Others

Q2. Number of bed of your hospital

Total number: _____beds

Number of general bed: _____beds

Number of bed for long-term care: _____beds

Q3. Number of patients per nurses based on the basic hospitalization fee standard

1. 7:1

2. 10:1

3. 13:1

4. 15:1

5. Others

Q4. Average length of stay in your hospital in March 2010: _____days

Q5. Type of your hospital (multiple answers)

1. Community care support hospital

2. Open access hospital

3. Special functioning hospital

4. Having beds for palliative care unit

5. Having beds for rehabilitation

6. Target hospital of Diagnosis Procedure Combination

Q6. Affiliated institution or agency of your hospital

1. Healthcare facility for the elderly

2. Welfare facilities for the elderly

3. Visiting nurse service station

4. Home helper station

5. Day care or day service

6. Group home for demented elderly

7. Care house

8. Care management agency

9. Community comprehensive support center

10. Health check center

11. Health promotion center

12. Others

*Q7-9. Omitted because the results were not used the analysis in this article*

**II. Discharge planning system in your hospital**

Q10. In your hospital, does the discharge planning department exist?

1. Yes

2. No, but now planning

3. No

Q10-subQ for respondent answering “yes”

1) Name of the department: ______

2) Establishment year: _______

3) The department’s position in your hospital

1. Clinical division

2. Nursing division

3. Administrative division

4. Under direct control of hospital director

5. Others

4) Service other than discharge planning (multiple answers)

1. Service about medical expenditure

2. Home care instructions for outpatients

3. Service about medical consultation

4. Visiting nurse

5. Responding to community healthcare workers

6. Others

5) Person in charge

1. Physician

2. Nurse

3. Medical Social Worker

4. Clerical staff

5. Others

6) Person in charge is full-time? Yes or no

7) Each staff’s number

1. Physician

2. Nurse (incl. public health nurse)

3. Medical social worker

4. Clerical staff

5. Others

*Q11-13 Omitted because the results were not used the analysis in this article*

**III Situation of discharge planning nurse**

*Omitted because the results were not used the analysis in this article*

Thank you for your cooperation.
